# Application of Continuous Improvement of Pre‐Examination Process Based on Evidence‐Based Nursing Concept in Acute Chest Pain of Adults

**DOI:** 10.1155/emmi/8401158

**Published:** 2026-05-11

**Authors:** Qiongyao Gan, Rongrong Yao

**Affiliations:** ^1^ Emergency Resuscitation Room (North), Ruijin Hospital, Shanghai Jiao Tong University School of Medicine, Shanghai, 200025, China, shsmu.edu.cn

**Keywords:** adult, chest pain, evidence-based nursing, patient satisfaction, triage

## Abstract

**Objective:**

To evaluate the feasibility and effectiveness of implementing a continuous improvement of the pre‐examination process based on evidence‐based nursing concept in the management of acute chest pain in adults.

**Methods:**

Based on the inclusion and exclusion criteria, the 92 cases of acute chest pain patients who aged ranging from 18 to 75 years old were admitted to Ruijin Hospital, Shanghai Jiao Tong University School of Medicine, from December 2022 to December 2024 and were allocated by admission date into the control group (*n* = 46) and the observation group (*n* = 46). The control group received routine pre‐examination and triage, while the observation group underwent continuous improvement of the pre‐examination process based on evidence‐based nursing concept. The waiting time, consultation time, triage duration, triage accuracy rate, misdiagnosis rate, missed diagnosis rate, revisit rate, complaint rate, rescue success rate, and patient satisfaction were compared between the two groups.

**Results:**

The waiting time, consultation time, and triage duration in the observation group ([4.45 ± 1.21] min, [2.08 ± 0.44] min, [1.56 ± 0.37] min) were shorter than the control group ([5.61 ± 1.08] min, [2.97 ± 0.25] min, [2.52 ± 0.42] min) (*t* = 4.851, 11.928, 11.632, *p* < 0.05). The triage accuracy rate in the observation group was higher than the control group (95.65% *vs* 80.43%, *χ* ^2^ = 5.059, *p* < 0.05). The misdiagnosis rate, missed diagnosis rate, and complaint rate in the observation group were (4.35%, 2.17%, 2.17%) lower than the control group (17.39%, 17.39%, 17.39%) (*χ* ^2^ = 4.039, 6.035, 6.035, *p* < 0.05). The rescue success rate and patient satisfaction in the observation group (97.83%, 95.56%) were higher than the control group (84.78%, 79.49%) (*χ* ^2^ = 4.929, 5.144, *p* < 0.05).

**Conclusion:**

The implementation of an evidence‐based continuous improvement program for the pre‐examination process was associated with enhanced triage accuracy, reduced diagnostic errors and complaints, and improved rescue outcomes and patient satisfaction in adult patients with acute chest pain. These findings support the feasibility and potential clinical value of integrating structured, evidence‐guided processes into emergency triage workflows.

## 1. Introduction

Acute chest pain in adults is a common and critical emergency with high mortality, posing major challenges to healthcare systems [1]. Globally, chest pain accounts for approximately 130 million emergency visits annually, with 20%–40% caused by life‐threatening cardiovascular events, and carries a 30‐day all‐cause mortality of 5.8% [2]. Associated symptoms, such as palpitations, dyspnea, and a sense of impending doom, often signal serious conditions including acute myocardial infarction, aortic dissection, pulmonary embolism, and tension pneumothorax, which require urgent intervention to prevent fatal outcomes [3]. Thus, improving the rapid and accurate identification and management of these patients remains a pressing medical priority.

Pre‐examination and triage is used by medical institutions to classify patients efficiently, ensuring timely care for severe cases and reducing pressure on emergency departments [4, 5]. However, the current triage for acute chest pain faces three main challenges: Firstly, the causes of chest pain are complex. The traditional triage model relies on nurses’ empirical judgment, resulting in a misdiagnosis rate as high as 20%–30% [6]. Secondly, the construction of “chest pain centers” recommended by international guidelines requires that electrocardiogram (ECG) examinations be completed within 10 min after the first medical contact. However, the current process takes an average of more than 15 min. More importantly, the annual number of patients received by the emergency departments of tertiary hospitals in our country has increased by an average of 12% annually. Against the backdrop of the nonsynchronous increase in human resources, it is urgently necessary to achieve precise classification through intelligent triage tools [7]. Therefore, optimizing the triage process to enhance its scientific nature and effectiveness has become a focus of current research. Evidence‐based nursing emphasizes starting from problems encountered in clinical practice and integrating scientific research findings with clinical expertise and experience, as well as patient needs, to promote the comprehensive application of both direct and indirect experience in practice [8]. This concept provides new insights for improving triage processes, namely, by collecting and analyzing best evidence and combining it with nurses’ professional judgments and patient needs to develop more scientific and reasonable triage protocols [9].

This study aims to evaluate the feasibility and effectiveness of implementing a continuously improved pre‐examination process based on the concept of evidence‐based nursing in adult patients with acute chest pain, in order to offer practical insights for optimizing emergency triage workflows and enhancing patient outcomes within a clinical setting. This has significant implications for advancing process‐driven quality improvement and promoting patient safety in acute care.

## 2. Research Object and Methods

### 2.1. Ethical Approval of the Research Protocol

The study has obtained ethical approval from the Institutional Review Committee of Ruijin Hospital, Shanghai Jiao Tong University School of Medicine. All participants have written informed consent.

### 2.2. Patients

A total of 92 cases of acute chest pain patients who were admitted to Ruijin Hospital, Shanghai Jiao Tong University School of Medicine from December 2022 to December 2024, were selected as the study subjects for a quasi‐experimental study. Inclusion criteria were as follows: Patients met the diagnosis criteria of acute chest pain, which refers to the pain, pressure sensation, constriction sensation, or other discomfort reported by the patient within the thoracic cavity. Its possible causes may include various disease spectra, such as those related to the circulatory system, respiratory system, digestive system, and musculoskeletal system [10]; patients whose ages ranged from 18 to 75 years old; and patients who were accompanied by at least one guardian. Exclusion criteria were as follows: Patients had some conditions, such as blurred consciousness and mental disorder; patients with malignant tumors and serious infections; and patients with incomplete clinical data. The 92 cases were divided into the control group and the observation group based on their admission time sequence, with 46 cases in each group. The recruitment and screening process of this study were as follows: From December 2022 to December 2024, all patients presenting to the Emergency Department of Ruijin Hospital, Shanghai Jiao Tong University School of Medicine, with acute chest pain were initially screened. The emergency department attending physician daily reviewed emergency triage records and conducted rapid assessments of suspected cases in accordance with the aforementioned inclusion and exclusion criteria. The entire process was verified by two independent reviewers. Ultimately, 92 eligible patients completed the screening process and were enrolled in the study.

### 2.3. Determination of Sample Size

**FIGURE 1:**
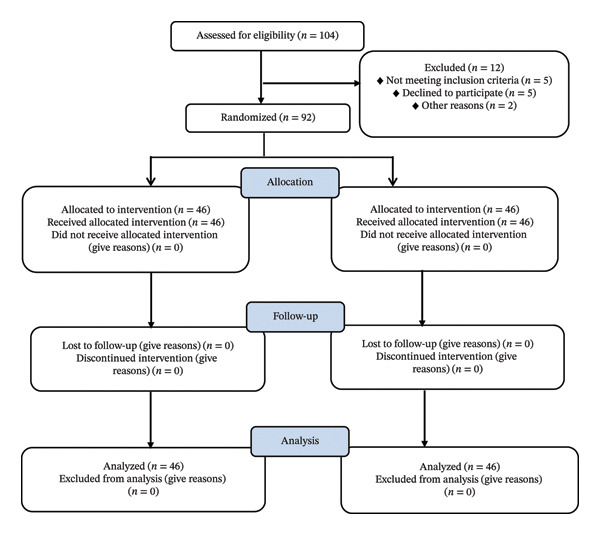
The flow diagram.

The sample size is determined according to the following formula: Control group sample size (n) =  ${\left[Z_{\alpha /2}\sqrt{p\left(1-p\left(1+c\right)/c\right)}+Z_{\beta }\sqrt{p_{1}\left(1-p_{1}\right)+p_{2}\left(1-p_{2}\right)/c}\right]}^{2}/{\left(p_{1}-p_{2}\right)}^{2}$, where *n* is the required sample size per group, *p*_1_ and *p*_2_ are the expected proportions in the two groups, *Z*_*α*/2_ and *Z*_*β*_are the critical values from the standard normal distribution for the type I and type II errors, respectively. *p*_1_ = 0.8, *p*_2_ = 0.5, *p* = (*p*_1_+*p*_2_)/2 = 0.65, *α* = 0.05, *β* = 0.10, *Z*_*α*/2_ = 1.96, *Z*_*β*_ = 1.282, *c* (distribution ratio) = 1, control group sample size (*n*) = observation group sample size (*n*) = 41, considering 10% error; the final sample size is determined to be 92. The flow diagram is shown in Figure 1.

### 2.4. Pre‐Examination and Triage Method

The control group received routine pre‐examination and triage, where nurses in charge of pre‐examination and triage utilized their clinical experience to sort patients based on their clinical signs. The specific process is carried out by nurses with more than 3 years of emergency care experience. Through inquiring about the patient’s chief complaint symptoms (such as the nature, duration, and radiation site of chest pain) and vital signs (blood pressure, heart rate, and blood oxygen saturation), a three‐grade classification is conducted: Grade I (Critical): typical manifestations of ST‐segment elevation myocardial infarction (persistent chest pain for more than 20 min + ST‐segment elevation on the arched back of the ECG); Grade II (emergency): non‐ST‐segment elevation acute coronary syndrome or aortic dissection (sudden tearing chest pain + bilateral blood pressure asymmetry); and Grade III (nonemergency): stable angina pectoris or noncardiogenic chest pain (such as gastroesophageal reflux disease). After making a judgment based on clinical experience, the nurse directly guides the patient to the corresponding treatment area (chest pain center, general emergency, or outpatient department), without the assistance of standardized tools for decision‐making.

The observation group received continuous improvement of the pre‐examination process based on evidence‐based nursing concept. The specific process is as follows: (1) Development and Application of the “Rapid Screening Form for Acute Chest Pain Chief Complaints”: Based on a review of current guidelines and evidence concerning life‐threatening causes of chest pain (e.g., acute coronary syndrome and aortic dissection), a structured screening form was developed to standardize initial nursing assessment. The form prompted nurses to systematically document five key clinical elements during triage: (a) nature of pain (e.g., squeezing/constricting); (b) radiation (e.g., to left arm/jaw); (c) relevant medical history (history of diabetes or hypertension); (d) duration of pain at rest (> 15 min); and (e) response to nitroglycerin. This form served as the foundational clinical decision support tool to guide subsequent triage actions. The Cronbach *α* of the rapid screening form was 0.82. To ensure consistency in assessment, a standardized 0–10 numeric rating scale (NRS) was mandated for pain intensity documentation across all cases. All triage nurses utilized the same hospital‐standardized 12‐lead ECG machine, and initial ECG interpretation was performed using the integrated, validated automated analysis software provided by the manufacturer. Nurses were trained to recognize critical automated alerts but were not required to provide independent, detailed interpretive reports. (2) Standardized Training and Competency Assessment for Triage Nurses: All triage nurses assigned to the chest pain clinic underwent a mandatory, standardized training program before implementation. The Training Covered: (a) The rationale and operational procedures for using the new rapid screening form; (b) The Updated Triage Workflow: Step 1: Immediate assessment of stability (airway, breathing, circulation, and mental status). Unstable patients were directed immediately to the resuscitation area per existing emergency protocols, bypassing the screening form. Step 2: For stable patients, the nurse initiated the “Rapid Screening Form.” The nurse verbally asked the patient the five structured questions and recorded the responses. Concurrently, a 12‐lead ECG was obtained. Step 3: The nurse evaluated the completed form and the ECG automated report. The presence of any one of the following criteria triggered assignment to the high‐priority “urgent evaluation” pathway: (i) pain nature suggestive of cardiac ischemia (e.g., constricting); (ii) radiation to left arm/jaw/back; (iii) history of coronary artery disease with pain > 15 min at rest; (iv) no relief with sublingual nitroglycerin (if taken); and (v) automated ECG report indicating “Acute MI Suspected” or “Significant ST‐T Abnormality.” Step 4: Patients meeting any high‐priority criterion were immediately escorted to a dedicated acute cardiac evaluation room. Patients not meeting these criteria were assigned to the standard waiting queue for further physician assessment. The completed screening form was attached to the patient’s chart for physician review. (c) Key clinical features and red flags for acute chest pain etiologies. Competency was assessed via a post‐training test. Only nurses who passed this assessment were authorized to implement the new process. (3) Multifaceted Process Optimization Interventions: The quality improvement program encompassed several parallel interventions to support the new triage protocol: (a) Physical and Informational Flow Optimization: Prominent signage outlining the new triage process was installed within the clinic. Informational posters were displayed to educate patients and caregivers. Additional guide nurses were stationed in high‐traffic areas to direct patient flow and provide initial assistance. (b) Ongoing Professional Development: Regular in‐service training sessions were conducted postimplementation on a bimonthly schedule to reinforce knowledge on acute chest pain management and professional communication skills. Each 60‐min session reviewed recent difficult cases, highlighted common errors in form completion, and provided updates on relevant guidelines. These sessions were supervised and evaluated by senior clinical staff. (c) Structured Feedback and Task Assignment System: A formal mechanism was established for nurses to report operational issues encountered during triage. Unresolved issues were escalated for departmental review. Specific process improvement tasks were assigned to nursing staff, with progress tracked and reported regularly. (4) Establishment of a Continuous Quality Improvement Framework: A formal Plan‐Do‐Study‐Act (PDSA) cycle was institutionalized. The implementation process for each PDSA cycle was as follows: In the plan phase, a specific, measurable aim was set (e.g., “reduce triage‐to‐ECG time to under 5 min for 90% of patients”). In the do phase, a change was implemented on a small scale (e.g., trialing a dedicated ECG technician in the triage area for 1 week). In the study phase, data were collected and analyzed against the aim. In the act phase, based on the analysis, the change was adopted, adapted, or abandoned. For example, an identified problem of triage delay was root‐cause analyzed and found to be related to occasional ECG machine malfunction. The countermeasure involved procuring backup equipment and creating a contingency plan. Monthly quality improvement meetings were held, with the triage accuracy rate (target ≥ 95%), defined as the percentage of patients correctly assigned to the high‐priority pathway based on final discharge diagnosis, serving as the key performance indicator. The triage process and supporting tools were dynamically adjusted based on data from these meetings and ongoing feedback.

### 2.5. Outcome Measures

The waiting time, consultation time, and triage time of the two groups were recorded, and the triage accuracy rate (calculated as the proportion of patients whose initial triage level matched the final diagnosis‐based acuity level. The reference standard for the final diagnosis was adjudicated by two senior emergency physicians based on comprehensive evaluation), misdiagnosis rate (defined as the proportion of patients who were assigned an incorrect specific diagnosis), missed diagnosis rate (defined as the proportion of patients in whom a life‐threatening condition was initially overlooked or not suspected during triage), revisit rate (defined as the proportion of patients who returned to the emergency department for chest pain or related symptoms within 72 h of discharge), complaint rate (defined as the proportion of patients or families who formally filed a complaint regarding the triage or initial management process), and rescue success rate (refers to the proportion of acute chest pain patients who are classified as level I or level II during the emergency triage and assessment, and after receiving initial emergency rescue and treatment, achieve hemodynamic stability and are safely transferred to the next stage of treatment) of two groups were statistically analyzed. The nursing satisfaction of two groups of patients was assessed using the satisfaction scale self‐made by our hospital. This scale consists of 10 items, each scored from 1 to 5 points, with a total score ranging from 10 to 50 points. A score of 50 was considered “*very satisfied*,” scores ranging from 40 to 49 were deemed “*satisfied*,” scores between 30 and 39 were rated as “*moderately satisfied*,” scores between 20 and 29 indicated “*dissatisfied*,” and scores from 10 to 19 were classified as “*very dissatisfied*.” The overall satisfaction was calculated based on the percentages of patients who were “*very satisfied*” and “*satisfied*.” The Cronbach *α* of the satisfaction scale was 0.84. The waiting time, consultation time, triage time, triage accuracy rate, misdiagnosis rate, and missed diagnosis rate are primary outcomes, and the remaining indicators are secondary outcomes.

### 2.6. Statistical Methods

The data analysis was conducted utilizing SPSS 24.0 software. Continuous variables with a normal distribution were represented as the mean ± standard deviation, and comparisons between groups were made using the independent‐samples *t*‐test. Categorical data were expressed as frequencies and percentages, with the chi‐square tests employed for comparisons between the groups. *p* < 0.05 was considered significant.

## 3. Results

### 3.1. Baseline Characteristics

There are no obvious differences in sex, age, body mass index, complications, smoking history, time from onset to hospital admission, visit periods, final diagnosis, initial triage level, and ECG findings between the two groups (*p* > 0.05), as shown in Table 1.

**TABLE 1:** Baseline characteristics of two groups.

Variable	Observation group (*n* = 46)	Control group (*n* = 46)	*t*/*χ* ^2^‐values	*p*‐values	*OR* (95%CI)
Sex (male/female)	28/18	32/14	0.767	0.381	0.681 (0.287–1.613)
Age (years)	52.37 ± 5.62	53.44 ± 6.79	0.823	0.413	
Body mass index (kg/m^2^)	23.73 ± 1.34	23.86 ± 1.57	0.427	0.670	

*Complications*					
Diabetes	5 (10.87)	7 (15.22)	0.383	0.536	0.679 (0.199–2.321)
Hypertension	10 (21.74)	8 (17.39)	0.276	0.599	1.319 (0.468–3.716)
Smoking history	12 (26.09)	14 (30.43)	0.214	0.643	0.807 (0.325–2.004)
Time from onset to hospital admission (h)	2.86 ± 0.62	2.79 ± 0.56	0.568	0.571	
Visit periods			0.425	0.514	0.753 (0.320–1.769)
Daytime	28 (60.87)	31 (67.39)			
Nighttime	18 (39.13)	15 (32.61)			

**Final diagnosis**					
**Life-threatening conditions**					

Acute myocardial infarction (AMI)	8 (17.39)	6 (13.04)	0.337	0.562	1.404 (0.445–4.423)
Unstable angina (UAP)	10 (21.74)	9 (19.57)	0.066	0.797	1.142 (0.416–3.138)

*Total ACS*					
Aortic dissection (AD)	2 (4.35)	1 (2.17)	‐	1.000	‐
Pulmonary embolism (PE)	1 (2.17)	2 (4.35)	‐	1.000	‐
Non–life‐threatening conditions					
Other cardiac causes	3 (6.52)	4 (8.70)	‐	1.000	‐
Noncardiac chest pain	22 (47.83)	24 (52.17)	0.174	0.677	0.840 (0.371–1.904)
Initial triage level			‐	0.726	‐
Level I (critical)	4 (8.70)	3 (6.52)			
Level II (emergency)	25 (54.35)	22 (47.83)			
Level III (urgent)	17 (36.96)	21 (45.65)			

*Key examination findings*					
Initial ECG suggestive of ischemia	17 (36.96)	15 (32.61)	0.192	0.662	1.211 (0.513–2.861)
Peak troponin positivity	16 (34.78)	13 (28.26)	0.453	0.501	1.354 (0.560–3.275)

*Note:* “‐” indicates that the result is Fisher’s exact probability.

### 3.2. The Pre‐Examination Efficiency

The waiting time, consultation time, and triage duration in the observation group were shorter than the control group; the triage accuracy rate in the observation group was higher than the control group (*p* < 0.05), as shown in Table 2.

**TABLE 2:** Comparison of pre‐examination efficiency between the two groups.

Groups	Waiting time (min)	Consultation time (min)	Triage duration (min)	The triage accuracy rate (*n*, %)
Observation group (*n* = 46)	4.45 ± 1.21	2.08 ± 0.44	1.56 ± 0.37	44 (95.65)
Control group (*n* = 46)	5.61 ± 1.08	2.97 ± 0.25	2.52 ± 0.42	31 (80.43)
*χ* ^2^/*t*‐values	4.851	11.928	11.632	5.059
*p*‐values	< 0.001	< 0.001	< 0.001	0.024

### 3.3. The Diagnosis and Treatment Quality

The misdiagnosis rate and missed diagnosis rate in the observation group were lower than the control group (*p* < 0.05), and the revisit rate between the two groups has no obvious differences (*p* > 0.05), as shown in Table 3.

**TABLE 3:** Comparison of diagnosis and treatment quality between the two groups (*n*, %).

Groups	Misdiagnosis rate	Missed diagnosis rate	Revisit rate
Observation group (*n* = 46)	2 (4.35)	1 (2.17)	0 (0.00)
Control group (*n* = 46)	8 (17.39)	8 (17.39)	4 (8.70)
*χ* ^2^‐values	4.039	6.035	‐
*p*‐values	0.044	0.030	0.117
OR (95% CI)	0.216 (0.043–1.020)	0.106 (0.013–0.882)	‐

*Note:* “‐” indicates that the result is Fisher’s exact probability.

### 3.4. The Rescue Success Rate and Complaint Rate

The rescue success rate in the observation group was higher than the control group, while the complaint rate was lower than the control group (*p* < 0.05), as shown in Table 4.

**TABLE 4:** Comparison of rescue success rate and complaint rate between the two groups (*n*, %).

Groups	Rescue success rate	Complaint rate
Observation group (*n* = 46)	45 (97.83)	1 (2.17)
Control group (*n* = 46)	39 (84.78)	8 (17.39)
*χ* ^2^‐values	4.929	6.035
*p*‐values	0.026	0.030
OR (95% CI)	8.077 (0.951–68.564)	0.106 (0.013–0.882)

### 3.5. The Patients’ Satisfaction

The patients’ satisfaction of the observation group was higher than the control group (*p* < 0.05), as shown in Table 5.

**TABLE 5:** Comparison of patients’ satisfaction between the two groups.

Groups	Very satisfied	Satisfied	Moderately satisfied	Dissatisfied	Very dissatisfied	Overall satisfaction
Observation group (*n* = 45)	23 (51.11)	20 (44.44)	1 (2.22)	1 (2.22)	0 (0.00)	43 (95.56)
Control group (*n* = 39)	15 (38.46)	16 (41.03)	5 (12.82)	2 (5.13)	1 (2.56)	31 (79.49)
*χ* ^2^‐values						5.144
*p*‐values						0.023
OR (95%CI)						5.548 (1.101–27.949)

*Note:* Seven deceased patients were excluded in the control group, and 1 deceased case was excluded in the observation group. Not all the deceased patients were included in the satisfaction analysis.

## 4. Discussions

Acute chest pain, as a common and potentially life‐threatening clinical symptom, necessitates prompt and accurate diagnosis and management for optimal patient prognosis [11, 12]. The etiology of acute chest pain is complex and diverse, encompassing cardiovascular, respiratory, digestive, and other systems, with some causes, such as acute myocardial infarction and aortic dissection carrying extremely high mortality and morbidity rates [13]. Consequently, pre‐examination, as the initial step in a patient’s healthcare journey, is of paramount importance [14]. Traditional pre‐examination protocols often rely on clinicians’ experience and intuition, and may have inherent limitations due to their subjective nature [15]. The application of evidence‐based nursing concept can significantly enhance the accuracy and efficiency of pre‐examination [16]. Therefore, this study aims to explore the application effect of the continuous improvement of the pre‐examination process based on evidence‐based nursing concepts in acute chest pain cases. This is of great clinical significance for optimizing the allocation of emergency resources, reducing the risk of missed diagnoses for critical cases, and enhancing the overall treatment efficiency of the chest pain center.

A previous study applied evidence‐based practice to pre‐examination and triage for patients with nontraumatic acute abdomen, and the results showed that this method could effectively improve the accuracy of patient triage and shorten the triage duration [17]. Another study applied continuous improvement in pre‐examination processes based on the evidence‐based nursing concept to pediatric outpatient clinics. The results showed that this pre‐examination process could effectively enhance pre‐examination efficiency in pediatric outpatient clinics and improve parents’ satisfaction [18]. Our quality improvement study observed that the observation group had shorter waiting time, consultation time, and triage duration compared to the control group, with a higher accuracy rate in triage. The results are consistent with earlier research and suggest that structuring triage workflows around scientific evidence may be associated with gains in efficiency and accuracy. Together, these studies support the potential value of evidence‐based concepts in refining pre‐examination workflows and enhancing the quality of medical services. The causes of acute chest pain are complex, which places extremely high demands on the timeliness and accuracy of triage. Therefore, the significant reductions observed in this study across three critical time intervals “waiting time, consultation time, and triage duration” may hold greater clinical significance compared to studies on acute abdominal pain or general pediatric outpatient settings. The observed improvements in triage efficiency might be explained by the core principle of evidence‐based nursing, which integrates the best available evidence with clinical expertise and patient circumstances to guide care plans [19]. Due to the optimization and standardization of the pre‐examination process, patients’ waiting times were significantly shortened. By optimizing the sequence and content of inquiry, doctors were able to obtain patients’ medical history and symptom information more quickly and accurately, which contributed to shortening the consultation time and improving the triage accuracy rate.

Furthermore, our study found lower rates of misdiagnosis, missed diagnosis, revisit, and complaints, alongside higher rescue success and patient satisfaction in the intervention group. A possible explanation is that the evidence‐based, continuous improvement model requires nursing staff to consistently evaluate outcomes, reflect on experience, and iteratively refine the process. This approach potentially fosters a deeper understanding and application of scientific evidence, enabling nurses to better integrate patient‐specific information and utilize reliable theoretical knowledge in their decision‐making [20]. Such a practice environment may contribute to fewer clinical decision errors and reduced diagnostic inaccuracies and ultimately be associated with enhanced patient satisfaction and fewer complaints.

## 5. Conclusions

The implementation of a continuously improved, evidence‐based pre‐examination process for adult patients with acute chest pain was associated with positive outcomes in this quality improvement study. The findings suggest potential benefits including enhanced pre‐examination efficiency, reduced rates of misdiagnosis, missed diagnosis, and complaints, as well as elevated patient rescue success rates and satisfaction. The operational framework and principles of this quality improvement program are potentially generalizable and adaptable to other emergency departments, particularly those in resource‐limited settings, aiming to standardize chest pain triage.

However, this study has several limitations that should be considered. First, the nonrandomized, time‐based allocation of patients (sequential admission) is a major methodological constraint. Although baseline characteristics were comparable, this quasi‐experimental design cannot fully eliminate the potential for selection bias and confounding due to temporal trends, such as seasonal variations in case mix or concurrent changes in hospital protocols. For instance, this study is a single‐center research with a limited sample size, which may not fully reflect the actual situation of all adult patients with acute chest pain. There is a lack of in‐depth research and analysis on the influencing factors of acute chest pain, and its clinical promotion is restricted. Second, the assessment of patient satisfaction remains a highly subjective outcome measure. Patients were not blinded to the intervention, and their perceptions may have been directly influenced by their experience with the new, more structured pre‐examination process, potentially inflating satisfaction scores in the observation group due to the Hawthorne effect or response bias. More critically, the satisfaction survey was inherently limited to surviving patients who could respond at discharge. The exclusion of deceased patients from this analysis, while methodologically unavoidable for a survey, introduces a significant source of nonresponse bias. It limits the generalizability of the satisfaction results and likely creates an over‐optimistic estimate of overall patient experience, as the perspectives of the most severe, and potentially dissatisfied, clinical outcomes are systematically missing. Future studies can conduct multicenter, prospective, and large‐sample studies, and deeply analyze the influencing factors of acute chest pain, to more comprehensively evaluate and optimize the application effect of the continuously improved pre‐examination process based on the concept of evidence‐based nursing in adult patients with acute chest pain. Although this study is a single‐center trial with a limited sample size, it provides a foundation and reference for future multicenter, large‐scale studies. Healthcare policymakers should encourage and support more evidence‐based nursing research to advance scientific inquiry and innovation in the medical field. By continuously improving the medical evidence system, we can establish a more robust scientific basis for healthcare policy formulation and drive progress in the medical industry. The intervention in this study involves the training of nursing staff and process reengineering, making it impossible to implement blinding for the triage nurses or the patients. This may introduce performance bias and expectation bias. We have adopted objective indicators based on timestamps from the electronic medical record system to minimize potential bias as much as possible.

## Author Contributions

Qiongyao Gan conceived and designed experiments; Qiongyao Gan performed experiments and data analysis; Qiongyao Gan and Rongrong Yao provided technical support, data collection, and analysis; and Qiongyao Gan wrote the manuscript.

## Funding

No funding was obtained for this study.

## Disclosure

All authors provided final approval for the submitted and published version.

## Ethics Statement

The study has obtained ethical approval from the Institutional Review Committee of Ruijin Hospital, Shanghai Jiao Tong University School of Medicine (approval no. 20220126). All participants have written informed consent.

## Consent

All of the authors have consented to publish this research.

## Conflicts of Interest

The authors declare no conflicts of interest.

## Data Availability

The data that support the findings of this study are available from the corresponding author upon reasonable request.
